# Peripheral Nerve Injury Induces Dynamic Changes of Tight Junction Components

**DOI:** 10.3389/fphys.2018.01519

**Published:** 2018-10-30

**Authors:** Xinghui Wang, Yang Miao, Jun Ni, Yaxian Wang, Tianmei Qian, Jun Yu, Qianyan Liu, Pan Wang, Sheng Yi

**Affiliations:** ^1^Key Laboratory of Neuroregeneration of Jiangsu and Ministry of Education, Co-innovation Center of Neuroregeneration, Nantong University, Nantong, China; ^2^Department of Pharmacy, Yancheng City No. 1 Peoples’ Hospital, Yancheng, China; ^3^Department of Rehabilitation Medicine, The Affiliated Hospital of Nantong University, Nantong, China

**Keywords:** peripheral nerve injury, tight junction, peripheral nerve barriers, bioinformatic analysis, matrix metalloproteinases

## Abstract

Tight junctions seal off physical barriers, regulate fluid and solute flow, and protect the endoneurial microenvironment of the peripheral nervous system. Physical barriers in the peripheral nervous system were disrupted after nerve injury. However, the dynamic changes of tight junction components after peripheral nerve injury have not been fully determined yet. In the current study, by using previously obtained deep sequencing outcomes and bioinformatic tools, we found that tight junction signaling pathway was activated after peripheral nerve injury. The investigation of the temporal expression patterns of components in tight junction signaling pathway suggested that many claudin family members were down-regulated after nerve injury. Moreover, we examined the effects of matrix metalloproteinases 7 and 9 (MMP7 and MMP9) on tight junction genes both *in vitro* and *in vivo* and found that MMP7 and MMP9 modulated the expressions of genes coding for claudin 1, claudin 10, and claudin 22. Our study revealed the dynamic changes of tight junction components after peripheral nerve injury and thus might contribute to the understanding of the molecular mechanisms underlying peripheral nerve injury and regeneration.

## Introduction

Peripheral nerves are protected by several physical barriers, such as endoneurial endothelial barrier, perineurial barrier, and Schwann cell barrier. These physical barriers, under physiological conditions, strictly regulate fluid and solute movement across the perineurium, shelter peripheral nerves from surrounding blood vessels, and thus help to generate an isolated region (Sano and Kanda, 2011; Weerasuriya and Mizisin, 2011). On the other hand, under pathological conditions such as peripheral neuropathies, physical barriers in the peripheral nervous system are damaged, vascular permeability is altered, and the endoneurial microenvironment is changed (Mizisin et al., 1990; Manole et al., 2015; Liu et al., 2018).

Physical barriers in the peripheral nervous system are mainly sealed by tight junction proteins claudins, occludin, and zona occluden (ZO) (Manole et al., 2015; Reinhold and Rittner, 2017). Claudins and occludin are transmembrane proteins that form hemophilic and/or heterophilic interactions and constitute the main components of tight junctions (Schneeberger and Lynch, 2004). ZO binds to claudins and occludin via protein-protein interaction domains and links claudins and occludin to the actin cytoskeleton (Forster, 2008). Assembled tight junction proteins help the formation and maintenance of cellular adhesive contacts and contribute to cellular organization.

It has been demonstrated that injury to the peripheral nervous system induces expression changes of tight junction proteins and compromises physical barriers (Hirakawa et al., 2003; Moreau et al., 2016; Reinhold and Rittner, 2017; Liu et al., 2018). Adhesive tight junctions and functional physical barriers provide necessary mechanical linkages and thus are important for nerve regeneration (Dezawa et al., 1996). However, only few studies focused on physical barriers in the peripheral nervous system and the molecular changes of tight junction components during peripheral nerve regeneration remain elusive.

Therefore, in our current study, by using rat sciatic nerve crush model, we observed the morphological changes of tight junctions, investigated the involvement of tight junction signaling pathway, and examined the temporal expression patterns of tight junction components. Furthermore, we studied the effects of metalloproteinases (MMPs) on tight junction genes and showed that altered physical barriers in the peripheral nervous system might be regulated by elevated MMPs.

## Materials and Methods

### Ethics Statement, Animal Surgery, and Tissue Collection

Sprague-Dawley (SD) rats were purchased from the Experimental Animal Center of Nantong University, Nantong, Jiangsu, China. All experimental and animal handling procedures were executed according to the Institutional Animal Care Guidelines of Nantong University and all animal experiments were ethically approved by the Administration Committee of Experimental Animal, Jiangsu, China.

Surgical procedures were performed as previously described (Yi et al., 2015). Male, adult SD rats were anesthetized by intraperitoneal injection with mixed narcotics containing 85 mg/kg trichloroacetaldehyde monohydrate, 42 mg/kg magnesium sulfate, and 17 mg/kg sodium pentobarbital. Skin incisions were performed on the mid-thigh of the left hind limbs. Rat sciatic nerves were exposed, lifted, and crushed with forceps for three times with 10 s each. At 1, 4, 7, and 14 days after surgery, rats were anesthetized and then sacrificed by decapitation. Sciatic nerve segments at the crush sites were collected and subjected to RNA extraction for deep sequencing and quantitative real-time PCR experiments. Normal sciatic nerve segments were also collected and used as controls.

### Transmission Electron Microscopy

Control SD rats or SD rats at 4 days after sciatic nerve crush were perfused with a fixative containing 1% paraformaldehyde and 1.25% glutaraldehyde. Sciatic nerve tissue specimens were harvested and fixed with 4% glutaraldehyde, post-fixed with 1% osmium tetroxide, washed, dehydrated, embedded in Epon 812 epoxy resin (Sigma-Aldrich, St. Louis, MO, United States), and cut into ultra-thin sections. Sciatic nerve sections were stained with lead citrate and uranyl acetate. Distal nerve segments were observed under a transmission electron microscope (JEOL Ltd., Tokyo, Japan) at magnifications of × 0.7 k, × 1.0 k, and × 30.0 k.

### Bioinformatic Analysis

Bioinformatic analysis was performed by using previously obtained deep sequencing outcomes (Yi et al., 2015). The expression levels of genes at 1, 4, 7, and 14 days after sciatic nerve crush were compared with their expressions in the control nerve segments. Genes with a fold change > 2 or < -2 and a false discover rate ≤ 0.001 were considered as differentially expressed and were subjected to canonical signaling pathway analysis with Ingenuity^®^ Pathway Analysis software (IPA^®^, Qiagen, Valencia, CA, United States). Genes in tight junction signaling pathway were further studied by a Venn diagram with the Venny 2.1.0 online software^1^ (Oliveros, 2007) to determine the logical relationships of differentially expressed genes at 1, 4, 7, and 14 days.

### Quantitative Real-Time PCR

A total number of 30 SD rats were used for quantitative real-time PCR. Total RNAs were isolated with Trizol reagent (Life Technologies, Carlsbad, CA, United States) and were reverse transcribed to cDNAs with Prime-Script reagent kit (TaKaRa, Dalian, Liaoning, China). cDNAs were amplified with SYBR Green Premix Ex Taq (TaKaRa) on an Applied Biosystems Stepone real-time PCR System (Applied Biosystems, Foster City, CA, United States). Relative quantifications of claudin 1 (CLDN1), claudin 10 (CLDN10), claudin 22 (CLDN22), claudin 19 (CLDN19), tumor necrosis factor (TNF), and CCAAT/enhancer-binding protein alpha (CEBPA) genes were calculated by using the ΔΔCt method with mitochondrial ribosomal protein L10 (MRPL10) as the reference gene (Wang et al., 2017). The quality of performed quantitative real-time PCR was validated by a single peak melt curve and a single band product in the DNA gel. The sequences of primer pairs were listed in Supplementary Table S1.

### Western Blot Analysis

A total number of 30 SD rats were used for Western blot analysis and sciatic nerve segments at the crush sites were subjected to protein extraction. Protein samples were isolated via direct homogenization and lysed in Laemmli buffer. Protein lysates were aspirated repeatedly through 25-gauge needles and centrifuged at 14,000 rpm for 10 min. Collected supernatants were mixed with β-mercaptoethanol, glycerin, and bromophenol-blue and were incubated at 95°C for 5 min. Equal amounts of protein samples were resolved on 10% SDS-PAGE and electrotransferred to polyvinylidene fluoride (PVDF) membranes (Millipore, Bedford, MA, United States). Membranes were blocked in 5% nonfat dry milk for 2 h at room temperature, probed with primary antibodies: anti-claudin-10 (1:400; ab66053, Abcam, Cambridge, MA, United States), anti-claudin-19 (1:400; sc-365967, Santa Cruz Biotechnology, Santa Cruz, CA, United States), or anti-GAPDH (1:1000; YM3215, ImmunoWay Biotechnology Company, Newark, DE, United States) at 4°C overnight, subsequently incubated with HRP-conjugated secondary antibodies (1:5000; Pierce, Rockford, IL, United States) for 1 h at room temperature, and then developed with enhanced chemiluminescence reagent (Pierce). The quantification of band intensity was performed by using UTHSCSA ImageTools (Department of Dental Diagnostic Science at the University of Texas Health Science Center, San Antonio, TX, United States).

### Schwann Cell Culture and Treatment

Schwann cells were isolated from the sciatic nerve segments of neonatal SD rats and treated with anti-Thy1.1 antibody (Sigma, St Louis, MO, United States) and rabbit complement (Sigma-Aldrich) as previously described (Yi et al., 2016). Purified Schwann cells were grown in Dulbecco’s modified eagle medium (DMEM; Gibco, Grand Island, NY, United States) supplemented 10% fetal bovine serum (FBS; Gibco) in a humidified 5% CO_2_ incubator at 37°C. Cultured Schwann cells were transfected with 100 nM MMP7 or MMP9 siRNA (Ribobio, Guangzhou, Guangdong, China) with Lipofectamine RNAiMAX transfection reagent (Invitrogen, Carlsbad, CA, United States). After 24 h of treatment, Schwann cells were isolated and quantitative real-time PCR were performed to determine the mRNA abundances of CLDN1, CLDN10, and CLDN22 in Schwann cells.

### *In vivo* treatment

A total number of 18 SD rats were divided into three groups and exposed to sciatic nerve crush injury as previously mentioned. Rats in each group received the injection of 10 μl of a 1:1 volume mixture of Matrigel (BD Biosciences, Billerica, MA, United States) with 100 nM human recombinant MMP7 protein (Merck Millipore, Darmstadt, Germany), Matrigel with 100 nM human recombinant MMP9 protein, or Matrigel with 0.9% sodium chloride, respectively, at the crush site immediately after sciatic nerve crush. Rats were sacrificed by decapitation at 4 days after treatment. Sciatic nerve segments (3-mm-long crushed part) were collected for subsequent quantitative real-time PCR to determine the mRNA abundances of CLDN1, CLDN10, and CLDN22.

### Statistical Analysis

Statistical methods and results were reported according to the SAMPL guideline (Lang and Altman, 2015). Summarized numerical results were shown as mean (SD). Data calculations, statistical analysis, and histograms were performed by using GraphPad Prism 6.0 (GraphPad Software, Inc., La Jolla, CA, United States). Gaussian distribution was assumed. Paired two-tailed student’s *t*-test was applied for comparisons of two sets of data. One-way analysis of variance (ANOVA) and Dunnett’s multiple comparisons test were applied for the comparisons of multiple sets of data. An alpha level of 0.05 was used to calculate *P*-values and determine the probability of Type I error. Calculated *P*-values were listed in Supplementary Table S2.

## Results

### Peripheral Nerve Injury Induced Tight Junction Disruptions

**FIGURE 1:**
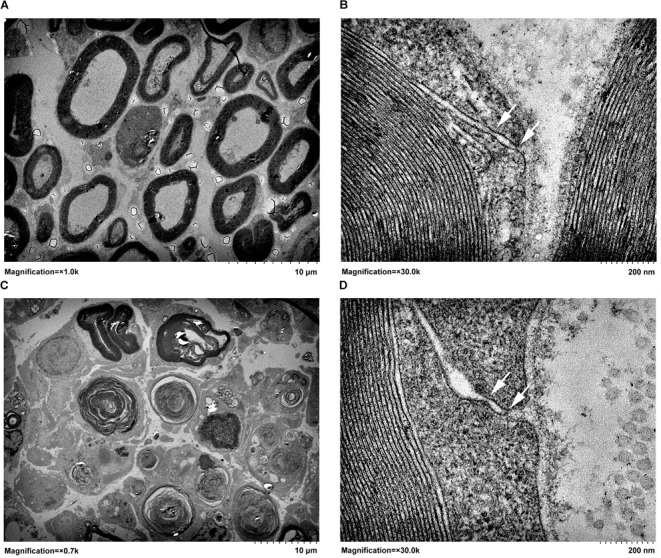
Transmission electron micrographs of **(A,B)** normal sciatic nerve segment and **(C,D)** sciatic nerve segment at 4 days after nerve crush. Arrows pointed to the kissing points of tight junctions. In **A** and **C**, magnifications were × 1.0 k and × 0.7 k, respectively, and the scale bar represented 10 μm. In **B and D**, magnification was × 30.0 k and the scale bar represented 200 nm.

The architectures of tight junctions in the distal sciatic nerve segments were detected by transmission electron microscopy. Thick myelin sheaths were observed in normal sciatic nerve segments (Figure 1A). Moreover, morphological images showed that tight junctions were observed in the outer mesaxons of myelinated axons. In normal rat sciatic nerve segments, tight junctions were visualized as a series of discrete sites of cell-cell kissing points (Figure 1B). Following injury to the peripheral nerves, the distal nerve segments undergo Wallerian degeneration. It was observed that the majority of the myelin sheath collapsed and disintegrated at 4 days after sciatic nerve crush. Only a few myelin sheaths did not degenerate and still existed (Figure 1C). The observation of the remaining myelin sheaths at a larger magnification showed that compared with uninjured normal nerves, the structure of tight junctions in injured nerve segments was less compact with larger cellular distances (Figure 1D).

### Peripheral Nerve Injury Activated Tight Junction Signaling Pathway

**FIGURE 2:**
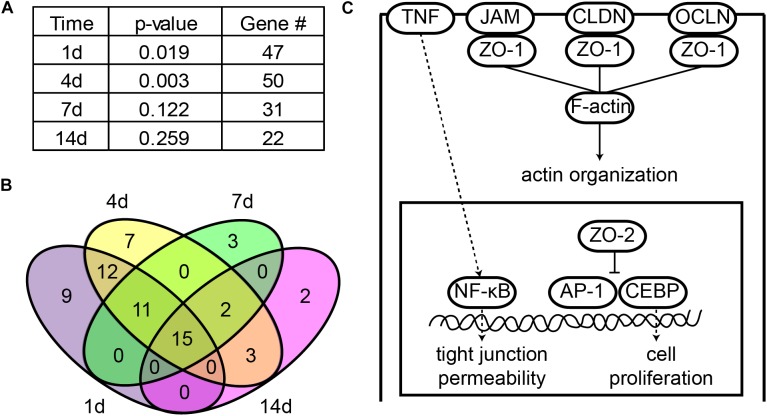
Tight junction signaling pathway was significantly involved following sciatic nerve crush. **(A)** Significance of tight junction signaling pathway at each time point after peripheral nerve injury. The -log (*P*-value) and number of differentially expressed genes at 1, 4, 7, and 14 days after nerve crush were listed. **(B)** Venn diagram of differentially expressed genes in tight junction signaling pathway. **(C)** The schematic diagram of tight junction signaling pathway. Signaling pathway diagram was modified from IPA canonical signaling pathway.

Given the fact that tight junctions were disrupted at early time points following peripheral nerve injury, we applied IPA canonical signaling pathway analysis to reveal whether tight junction signaling pathway was involved after nerve injury. IPA analysis showed that at 1 day after nerve injury, the *p-value* of tight junction signaling pathway was less than 0.02, suggesting that tight junction signaling pathway was significantly activated at the acute phase of peripheral nerve injury. Tight junction signaling pathway became more significantly involved at 4 days after peripheral nerve injury but less significantly involved at later time points (7 and 14 days) (Figure 2A).

All differentially expressed genes in this signaling pathway were identified (Supplementary Table S3) and the numbers of these differentially expressed genes at different time points were counted. Consistent with the trend of the significance of tight junction signaling pathway, a large number of genes were differentially expressed at early time points (1 and 4 days) while the number of differentially expressed genes decreased at later time points (7 and 14 days) (Figure 2A). Among these differentially expressed genes, 15 genes were commonly up-regulated or down-regulated at all time points after sciatic nerve injury (Figure 2B).

A schematic diagram of tight junction signaling pathway was obtained and modified from IPA database to show critical molecules in this signaling pathway (Figure 2C). It was demonstrated that occludin (OCLN), claudin (CLDN), and junctional adhesion molecule (JAM) clustered tight junction-associated protein ZO-1, ZO-2, and ZO-3, formed strands, linked to the actin cytoskeleton (F-actin), and regulated actin organization. ZO-2 also entered into the nucleus, inhibited transcription factors activator protein 1 (AP-1) and CCAAT/enhancer-binding protein (CEBP), and regulated cell proliferation. Meanwhile, the permeability of tight junctions was also regulated by cytokines, e.g., the pro-inflammatory cytokine TNF.

### Tight Junction Genes and Proteins Were Altered by Peripheral Nerve Injury

**FIGURE 3:**
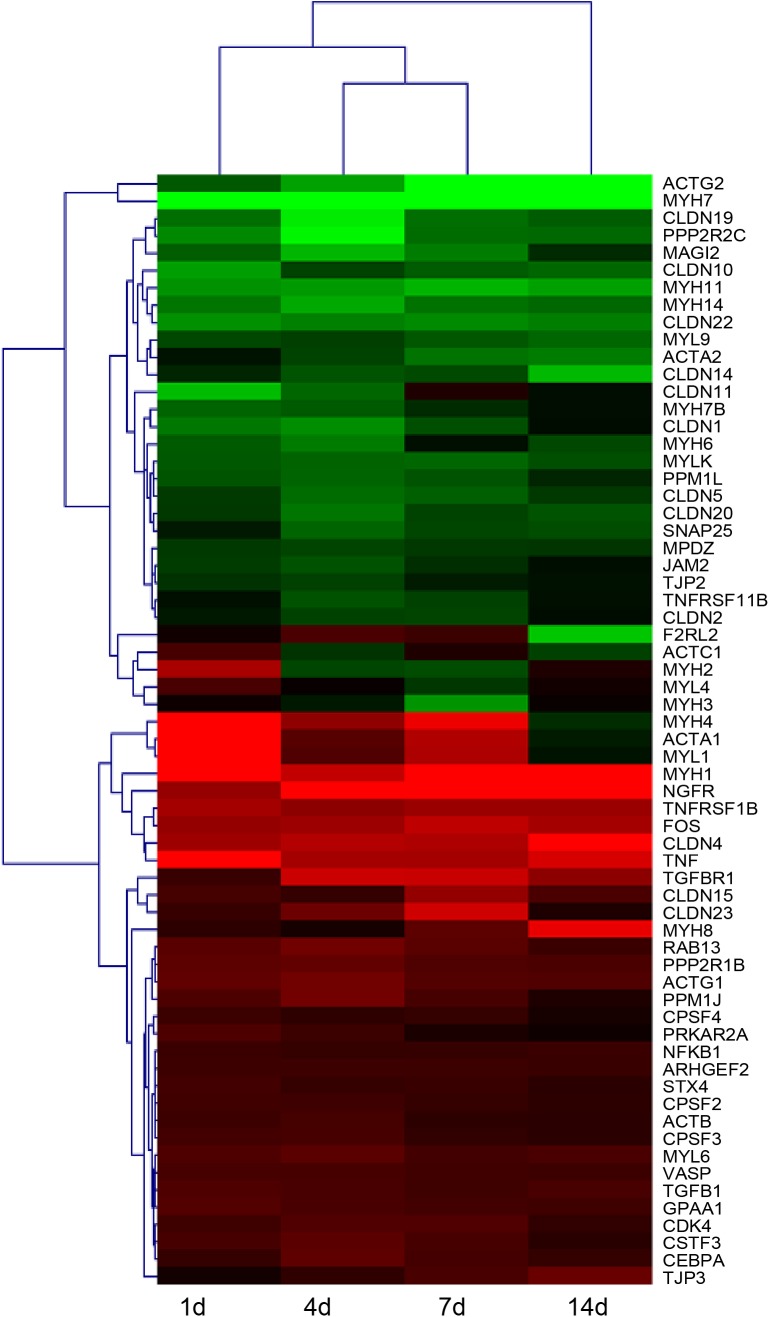
Heatmap and hierarchical clustering of differentially expressed genes in tight junction signaling pathway in sciatic nerve segments at 1, 4, 7, and 14 days after nerve crush. Gene expression levels at 1, 4, 7, and 14 days were compared with their expression levels in normal nerve segments and were indicated by the color bar. Up-regulated genes were labeled in red while down-regulated genes were labeled in green.

**FIGURE 4:**
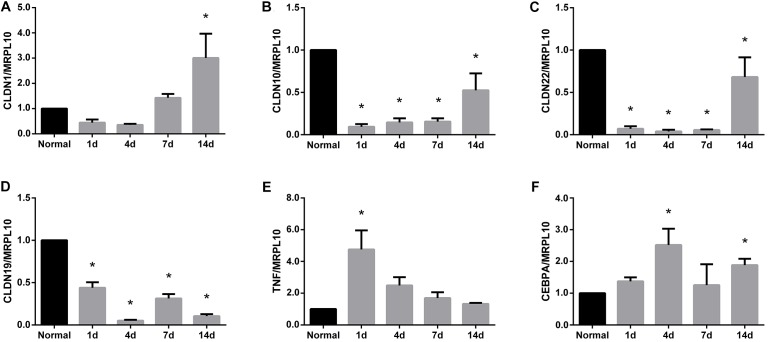
Quantitative real-time PCR analysis of the expression patterns of representative genes in tight junction signaling pathway. Gene expression patterns of **(A)** CLDN1, **(B)** CLDN10, **(C)** CLDN22, **(D)** CLDN19, **(E)** TNF, and **(F)** CEBPA were determined by quantitative real time PCR and were normalized to MRPL10. Numerical results were shown as mean (SD). ^∗^Statistically different from normal control (*n* = 3, Dunnett’s multiple comparisons test, *P* < 0.05).

After identifying that tight junction signaling pathway was activated after peripheral nerve injury, the temporal expression patterns of differentially expressed genes in this signaling pathway were determined and shown in a heatmap (Figure 3). Gene expression analysis showed that among 64 differentially expressed genes, approximately half of genes were up-regulated and half of genes were down-regulated. The accuracy of deep sequencing outcomes were further validated by quantitative real-time PCR. Considering that claudins are very critical for the formation of tight junctions (Lapierre, 2000; Angelow et al., 2008), we measured the expression levels of genes coding for members of the claudin family. Some other representative genes in the tight junction signaling pathway were also determined. Results from quantitative real-time PCR suggested that, in consistent with deep sequencing results, claudin family genes (CLDN1, CLDN10, CLDN22, and CLDN19) were down-regulated after sciatic nerve injury while TNF and CEBPA were up-regulated after nerve injury (Figure 4).

**FIGURE 5:**
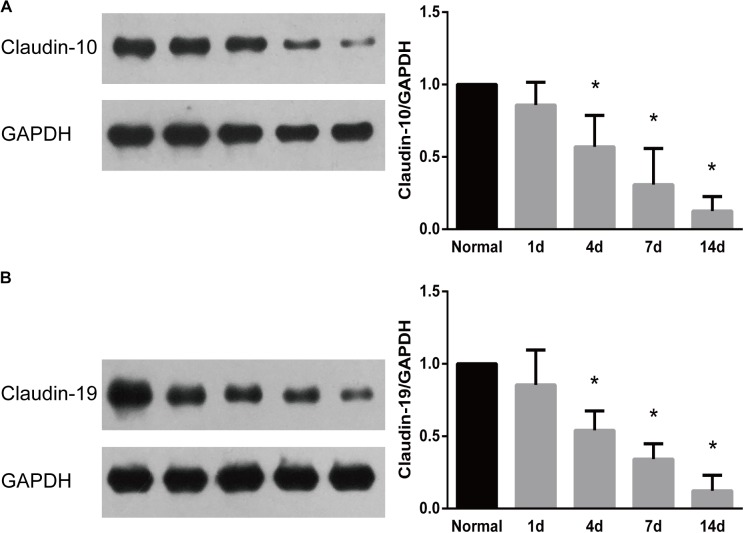
Western blot analysis of the expression patterns of representative proteins in tight junction signaling pathway. Protein expression patterns of **(A)** claudin 10 and **(B)** claudin-19 were determined by Western blot and normalized to GAPDH. Numerical results were shown as mean (SD). ^∗^Statistically different from 0 day control (*n* = 3, Dunnett’s multiple comparisons test, *P* < 0.05).

The temporal expression patterns of representative proteins were further examined by Western blots. Results from Western blots demonstrated that consistent with their temporal gene expressions, the protein expressions of both claudin-10 and claudin-19 were decreased after peripheral nerve injury (Figure 5). Decreased amounts of claudins might thus lead to disruption of tight junctions and the leakage of physical barriers.

### Tight Junction Gene Expressions Were Regulated by MMPs

Further studies were performed to determine whether these dysregulated tight junction genes could be modulated by MMPs. Since Schwann cells are the major cell population in the sciatic nerve segments, we cultured primary Schwann cells, transfected Schwann cells with MMP siRNAs, and measured the expression levels of tight junction genes in transfected cells.

**FIGURE 6:**
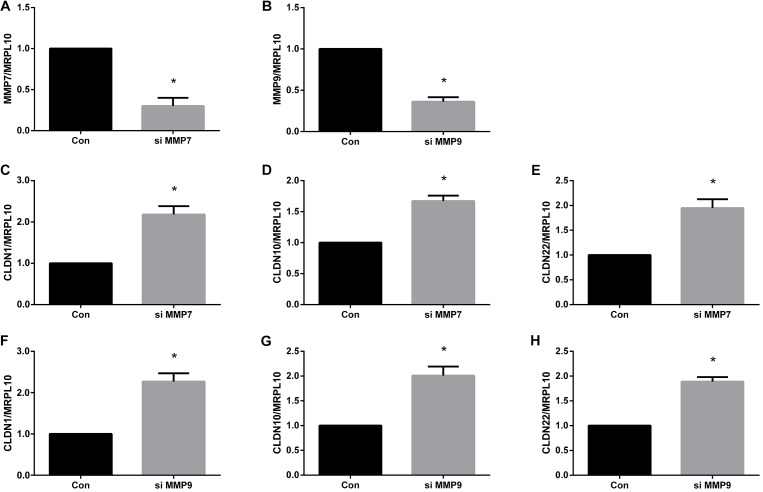
Quantitative real-time PCR analysis of tight junction genes in Schwann cells treated with MMPs. **(A)** MMP7 mRNA expression in Schwann cells transfected with MMP7 siRNA. **(B)** MMP9 mRNA expression in Schwann cells transfected with MMP9 siRNA. **(C–E)** The mRNA expression levels of **(C)** CLDN1, **(D)** CLDN10, and **(E)** CLDN22 in Schwann cells transfected with MMP7 siRNA. **(F–H)** The mRNA expression levels of **(F)** CLDN1, **(G)** CLDN10, and **(H)** CLDN22 in Schwann cells transfected with MMP9 siRNA. The expression levels of tight junction genes were expressed as relative abundance of target genes normalized to MRPL10 with respect to control. Numerical results were shown as mean (SD). ^∗^Statistically different from control (*n* = 3, paired *t*-test, *P* < 0.05).

Outcomes from quantitative real-time PCR showed that both MMP7 siRNA and MMP9 siRNA significantly down-regulated gene expressions of MMP7 and MMP9, respectively (Figures 6A,B). These siRNAs with high gene-silencing efficiency were then used for subsequent experiments. Quantitative real-time PCR results showed that the gene expression levels of CLDN1 (Figure 6C), CLDN10 (Figure 6D), and CLDN22 (Figure 6E) were higher in Schwann cells transfected with MMP7 siRNA compared with in cells transfected with siRNA control. Similarly, cells transfected with MMP9 siRNA also showed higher expression levels of CLDN1 (Figure 6F), CLDN10 (Figure 6G), and CLDN22 (Figure 6H).

### MMPs Regulated Tight Junction Genes *in vivo*

**FIGURE 7:**
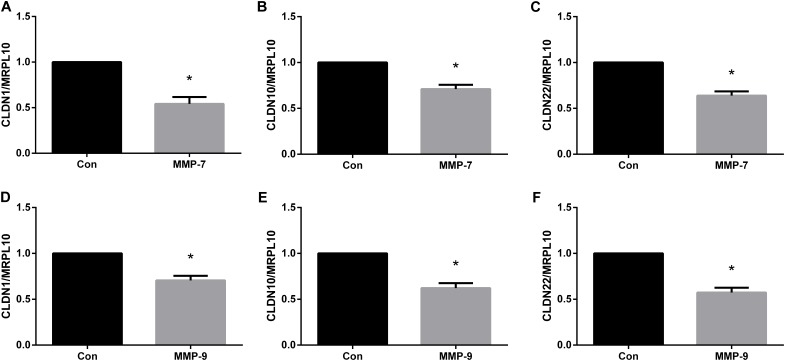
Quantitative real-time PCR analysis of tight junction genes in the injured sciatic nerve segments injected with MMPs. **(A–C)** The mRNA expression levels of **(A)** CLDN1, **(B)** CLDN10, and **(C)** CLDN22 at sciatic nerve crush site treated with human recombinant MMP7 protein. **(D–F)** The mRNA expression levels of **(D)** CLDN1, **(E)** CLDN10, and **(F)** CLDN22 at sciatic nerve crush site treated with human recombinant MMP9 protein. The expression levels of tight junction genes were expressed as relative abundance of target genes normalized to MRPL10 with respect to control. Numerical results were shown as mean (SD). ^∗^Statistically different from control (*n* = 3, paired *t*-test, *P* < 0.05).

In addition to *in vitro* studies, we used an animal model of peripheral nerve crush injury to investigate the *in vivo* effects of MMP7 or MMP9 on tight junction genes. As compared to the sodium chloride control group, the application of human recombinant MMP7 protein significantly decreased the abundances of tight junction genes CLDN1 (Figure 7A), CLDN10 (Figure 7B), and CLDN22 (Figure 7C) at 4 days after sciatic nerve injury. Similarly, the injection of human recombinant MMP9 protein also led to reduced mRNA expressions of CLDN1 (Figure 7D), CLDN10 (Figure 7E), and CLDN22 (Figure 7F). These results suggested that MMPs significantly reduced the amounts of tight junction genes *in vivo*.

## Discussion

Tight junctions constitute physical barriers in the peripheral nervous system and protect peripheral nerves from noxious surrounding stimulus (Reinhold and Rittner, 2017). Despite their important roles, the structure, function, and even molecular compositions of tight junctions in the peripheral nervous system have not been fully elucidated (Peltonen et al., 2013). The presences of tight junction proteins claudin-1, -2, -3, -5, -19, occludin, tricellulin and scaffolding proteins ZO-1, Disks Lost-multi PDZ domain protein 1, Pals-associated tight junction protein in the blood-nerve barrier of the peripheral nervous system have been identified previously (Poliak et al., 2002; Hirakawa et al., 2003; Pummi et al., 2004; Miyamoto et al., 2005; Alanne et al., 2009; Sauer et al., 2014). The wide application of high-throughput analysis largely helps the discovery of tight junction components. The bioinformatic analysis of microarray data, for instance, demonstrated the presence of claudin-14 and claudin-19 in rat distal nerve segments and suggested that these two tight junction proteins might be two central regulatory factors for Wallerian degeneration (Li et al., 2013). In this study, by using previously obtained deep sequencing outcomes, we found that there existed many tight junction genes in rat sciatic nerve segments, including 22 isoforms of CLDN (CLDN1, 2, 3, 4, 5, 6, 7, 8, 9, 10, 11, 12, 14, 15, 16, 17, 18, 19, 20, 22, 23, and 24), OCLN, TJP1 (tight junction protein 1, ZO-1), TJP2 (tight junction protein 2, ZO-2), and TJP3 (tight junction protein 3, ZO-3).

Besides revealing the presence of these tight junction genes, we also systematically studied the expression patterns of genes in the tight junction signaling pathway and found that 12 isoforms of claudins (CLDN1, 2, 4, 5, 10, 11, 14, 15, 19, 20, 22, 23) and 2 genes in the ZOs family (TJP2 and TJP3) were differentially expressed in at least one time point after sciatic nerve crush. The expressions of the majority of claudin isoforms (CLDN1, 2, 5, 10, 11, 14, 19, 20, and 22) were decreased after nerve injury while the expressions of a few isoforms (CLDN4, 15, and 23) were increased. Our observations were in consistent with preceding study which showed that claudin-1 and claudin-5 proteins were down-regulated and even absent after rat sciatic nerve ligation (Hirakawa et al., 2003). However, a previous study showed that CLDN14 was up-regulated in the distal nerve segments at 4 days after sciatic nerve transection (Gong et al., 2014). These results seems to be contradictory with our observations since our data showed that CLDN14 was kept down-regulated in the sciatic nerve segments at 1, 4, 7, and 14 days after sciatic nerve crush. It was shown that peripheral inflammation and peripheral nerve injury would induce tight junction disruption at different sites (Sauer et al., 2014). Different types of peripheral injuries might also lead to tight junction disruption at different locates. And this inconsistency might be due to the difference in nerve tissue segments (distal site versus injury site).

Quantitative real-time PCR was conducted to validate deep sequencing outcomes. Real-time PCR results showed that the mRNA expressions of claudin family members, including CLDN1, CLDN10, CLDN19, and CLDN22, were drastically down-regulated after peripheral nerve injury. Here, we used MRPL10 instead of ACTB or GAPDH, two commonly used internal controls, as the reference gene. ACTB is differentially expressed in the sciatic nerve stumps after rat peripheral nerve injury (Wang et al., 2018). GAPDH is a major enzyme involved in energy metabolism and expresses at different levels in damaged tissues and organs (Ghafoory et al., 2013). On the contrast, the mRNA expression levels of MRPL10 in rat sciatic nerve stumps were demonstrated to be relatively stable (Wang et al., 2017). Therefore, in the current study, we used MRPL10 as an internal control, calculated the relative abundances of CLDN1, CLDN10, CLDN19, CLDN22, TNF, and CEBPA at 1, 4, 7, and 14 days post nerve injury, and compared the expressions of these target genes with their expressions in the sciatic nerve stumps in normal rats. It is worth noting that compared with normal rats, rats that undergo comparable surgery (sham surgery) without crushing along the same time points, are more suitable controls for the determination of the temporal expression patterns of target genes and proteins.

Western blots outcomes also suggested that protein expressions of claudin-10 and claudin-19 were down-regulated after peripheral nerve injury. Claudins are critical tight junction proteins that exhibit vital barrier functions (Angelow et al., 2008). In the central nervous system, it was reported that loss of claudin-3 was correlated with the leakiness of the blood-brain barrier while expression of claudin-1 induced sealing of the blood-brain barrier (Pfeiffer et al., 2011). Therefore, we speculated that down-regulated claudins (and other tight junction proteins) might significantly disrupt tight junctions and lead to the leakage of physical barriers in the peripheral nervous system. Our current study, as far as we know, is the first systemical analysis of the dynamic changes of tight junction components following peripheral nerve injury. Future studies will be performed to further determine the expressions and localizations of tight junction proteins to obtain a more comprehensive physiological understanding.

Moreover, in the current study, we also explored the potential mechanisms that might account for the interruption of physical barriers in the peripheral nervous system. MMP9 has been demonstrated as a key factor that regulates barrier tightness (Yang et al., 2007; Hackel et al., 2012a,b). Notably, MMP-9 was highly elevated after sciatic nerve injury (Yu et al., 2016). The expression levels of another zinc-metalloproteinase, MMP-7, was also strongly increased (Qin et al., 2016). Therefore, we tested the hypothesis that up-regulated MMPs might mediate tight junction disruption by transfecting Schwann cells with MMP siRNAs. MMP7 and MMP9 siRNA transfection increased the mRNA expression levels of CLDN1, CLDN10, and CLDN22. 10 nM of recombinant MMP7 or MMP9 protein were directly added to Schwann cells as well. However, at this certain concentration of MMP7 or MMP9, no robust changes of the mRNA expressions of CLDN1, CLDN10, or CLDN22 were observed (data not shown). A higher concentration of recombinant MMP proteins may be needed to elicit changes of tight junction genes. The *in vivo* effects of MMP7 and MMP9 were also determined. It was observed that the expression levels of CLDN1, CLDN10, and CLDN22 were robustly reduced following the injection of MMP7 or MMP9 protein. These results collectively demonstrated that elevated MMPs might modulate the expressions of tight junction genes and provided a preliminary mechanism of the leakage of physical barriers after peripheral nerve injury.

In the peripheral nervous system, MMPs have been demonstrated to affect the migration of Schwann cells. The application of recombinant MMP9 protein significantly elevated the migration rate of Schwann cells (Mantuano et al., 2008). Our preliminary research also suggested that MMP7 and MMP9 could promote Schwann cell migration (unpublished data). Meanwhile, many molecules also affect Schwann cell migration via MMPs. For example, the promoting effect of uridine 5′-triphosphate on Schwannoma cell migration was through the activation of MMP2 (Lamarca et al., 2014). The enhancing role of protocatechuic acid on RSC96 Schwann cell migration was through the activation of MMP2 and MMP9 (Ju et al., 2015). On the other hand, the migration of cells may be modulated by tight junction proteins or factors that affect tight junctions. Occludin and ZO-1 were demonstrated to regulate the migration of epithelial cells and fibroblast-like cells, respectively (Du et al., 2010; Huo et al., 2011). Tumor necrosis factor receptor-associated factor 4, a negative regulator of tight junctions, was able to increase the migration of breast cancer cells (Rousseau et al., 2013). Therefore, it is possible that the promoting effects of MMP7 and MMP9 on Schwann cell migration are through modulating tight junctions in Schwann cells. However, besides the inhibitory effects of MMP7 and MMP9 on tight junction components, the direct effects of MMP7 and MMP9 on the functional recovery of injured peripheral nerves remain largely undetermined. Electrophysical examinations and functional tests, such as compound muscle actin potential (CMAP) recording and Catwalk gait analysis, should be conducted to determine whether the treatment of MMP7 or MMP9 would promote peripheral nerve repair and regeneration.

Notably, besides barriers in the Schwann cells, endoneurial endothelial barrier and perineurial barrier are also important physical barriers in the peripheral nervous systems. In the current study, by using deep sequencing, we found that tight junction components in sciatic nerve stumps were differentially expressed. Considering that Schwann cell constitute a main source of cells in sciatic nerves, here, we focused on tight junctions in the Schwann cells. But tight junctions in endoneurial endothelium and perineurium might also be altered after peripheral nerve injury since sequencing analysis examined the whole nerve tissue. The method of single cell sequencing may help to further identify changes of tight junction genes in Schwann cells, endoneurial endothelial cells, and perineurial cells.

Taken together, in our current study, with the joint use of deep sequencing data and bioinformatic analysis methods, we studied the significance of tight junction signaling pathway during peripheral nerve injury and regeneration, examined the dynamic molecular changes of tight junction components, and analyzed the mechanisms underlying changes of tight junction components. Our study helps to develop the understanding of the compositions and dynamic changes of tight junctions in physical barriers of the peripheral nervous system and may expand our knowledge about peripheral nerve injury and regeneration.

## Author Contributions

SY conceived and designed the experiments. XW, YM, JN, YW, TQ, JY, QL, PW, and SY performed the experiments. XW, YM, and SY analyzed the data. SY contributed to reagents, materials, and analysis tools. XW, JN, and SY wrote the manuscript.

## Conflict of Interest Statement

The authors declare that the research was conducted in the absence of any commercial or financial relationships that could be construed as a potential conflict of interest.

## Footnotes

1 http://bioinfogp.cnb.csic.es/tools/venny/index.html

## Supplementary Material

TABLE S1 Primer pairs for quantitative real-time PCR. The sequences of primers of target genes CLDN1, CLDN10, CLDN22, CLDN19, TNF, CEBPA, and reference gene MRPL10 were listed.

TABLE S2 Calculated *P*-values in histograms.

TABLE S3 The relative expression levels of differentially expressed genes in tight junction signaling pathway. The expression levels of genes in tight junction signaling pathway at 1, 4, 7, and 14 days after sciatic nerve crush were compared with those in normal nerve segments. Differentially expressed genes were listed and presented as log_2_Ratio.
